# Optimal Cytoplasmic Transport in Viral Infections

**DOI:** 10.1371/journal.pone.0008165

**Published:** 2009-12-30

**Authors:** Maria R. D'Orsogna, Tom Chou

**Affiliations:** 1 Department of Mathematics, California State University Northridge, Los Angeles, California, United States of America; 2 Department of Biomathematics and Department of Mathematics, University of California Los Angeles, Los Angeles, California, United States of America; BMSI-A*STAR, Singapore

## Abstract

For many viruses, the ability to infect eukaryotic cells depends on their transport through the cytoplasm and across the nuclear membrane of the host cell. During this journey, viral contents are biochemically processed into complexes capable of both nuclear penetration and genomic integration. We develop a stochastic model of viral entry that incorporates all relevant aspects of transport, including convection along microtubules, biochemical conversion, degradation, and nuclear entry. Analysis of the nuclear infection probabilities in terms of the transport velocity, degradation, and biochemical conversion rates shows how certain values of key parameters can maximize the nuclear entry probability of the viral material. The existence of such “optimal” infection scenarios depends on the details of the biochemical conversion process and implies potentially counterintuitive effects in viral infection, suggesting new avenues for antiviral treatment. Such optimal parameter values provide a plausible transport-based explanation of the action of restriction factors and of experimentally observed optimal capsid stability. Finally, we propose a new interpretation of how genetic mutations unrelated to the mechanism of drug action may nonetheless confer novel types of overall drug resistance.

## Introduction

In order to reproduce, viruses must exploit the internal machinery of host cells to synthesize key proteins and assemble new virions. The genetic material of membrane-enveloped viruses is contained within an internal protein capsid enclosed by a lipid membrane. Upon contact with a cell, complex interactions between cellular surface receptors and viral spike proteins [1], [2] induce fusion between viral and host cell membranes, allowing the protein capsid to enter the cell cytoplasm. For many viral species, the genome must also penetrate the nucleus and integrate with the host DNA. Some viruses wait for dissolution of the cell nuclear membrane during mitosis for genomic integration; others take a more active approach by directly transferring their RNA or DNA through nuclear pores. This infection mechanism allows viral reproduction at any stage of the cell cycle, and is utilized by lentiviruses such as HIV [3]–[5].

Getting to the nucleus from the cell periphery is a treacherous journey since viruses must navigate the cytoplasm, a crowded environment where diffusion is inhibited [6]–[8] and degradation may take place [9], [10]. Moreover, a series of biochemical steps, such as capsid disassembly and reverse transcription, must occur so that viral material is transformed into complexes that are able to enter the nucleus and integrate with the host genome.

The post-entry dynamics of HIV is especially complex since HIV capsids rapidly disassemble and give rise to intermediate structures that are difficult to visualize, even with modern fluorescent probe tracking techniques [11]. One of the capsid derivatives is the reverse transcription complex (RTC), through which RNA is processed into DNA while traveling to the nucleus [5]–[9]. Although the exact mechanisms are unknown, during its journey, the RTC sheds some of its proteins and acquires others. Ultimately, a preintegration complex (PIC), capable of nuclear entry, is formed [5], [7], [9].

Two key features of nuclear entry are thus: i) the microtubule assisted directional motion, and ii) the sequence of transformations from capsid to preintegration complex. Recent experiments using fluorescence imaging have revealed that cytoplasmic transport and viral transformation occur along microtubules, assisted by microtubule-associated molecular motors of the host cell [12]–[15]. Theoretical studies of viral transport in the cytoplasm include that of Lagache *et al.* [16], who studied viral transport as a microtubule-mediated convection, punctuated by free cytoplasmic diffusion. In their work, the motion of a single viral particle can be reduced to an effective drift, where the normalized velocity depends on molecular motor speeds, microtubule density, and viral detachment and attachment rates on the microtubule.

Biochemical transformations of the viral material are known to also play a critical role in determining the probability and timing of productive infections. Experiments that enhance or hinder specific viral transformations in the cytoplasm show that artificial delays or accelerations can result in abortive infections [9], [17]–[22]. In particular, various TRIM5 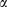 proteins that accelerate capsid disassembly appear to *inhibit* infection, providing *e.g.*, protection for humans against SIV [17], [18]. Entry failure may be due to increased cytoplasmic decay of the viral material, to biochemical constraints blocking nuclear entry, or to biochemical deficiencies that prevent integration with the host DNA. The emerging consensus is that biochemical transformations and transport must be balanced before successful infection can occur [23], [24]. However, there is no theoretical model showing how acceleration of transformations might reduce viral infection. Furthermore, although several authors have considered viral trafficking and microtubular transport [16], [25]–[27], the coupling between transport and biochemical transformations of viral material, and how this can enhance or suppress infection probabilities has remained largely unexplored.

In this paper, we tackle these questions through a stochastic model where viral entry depends on the interplay between physical transport along microtubules and the required serial biochemical transformations of the viral material, as observed in numerous studies [7]–[9]. The mathematical model relies on many simplifying assumptions, but captures the main steps involved in nuclear entry. One important consequence of this cytoplasmic transport model of viral infection is the emergence of optimal values of key parameters that the maximize nuclear entry probability, and consequently the productive infection efficiency. We analyze the probability and timing of nuclear entry, providing a possible explanation and quantitative framework for recent discoveries of an optimal capsid stability for HIV infections [17], [19], [20], [23]. Our findings also suggest a broader mechanistic interpretation of antiviral drug efficacy and resistance.

## Methods

**Figure 1:**
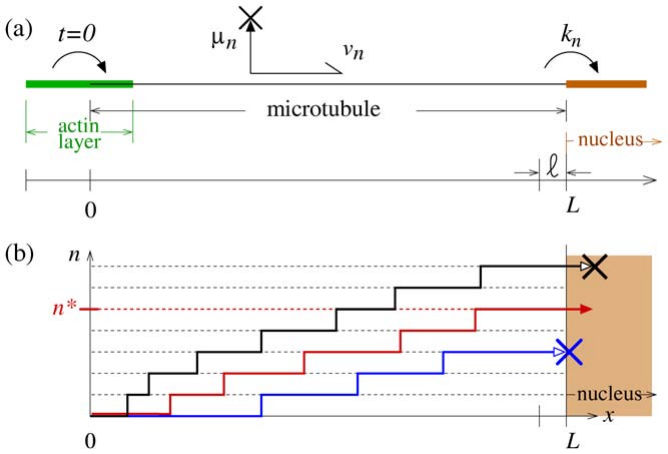
Schematic of one-dimensional cytoplasmic transport along a microtubule. Virus capsids, after entering the cell and passing through the actin rich cortical region, attach to molecular motors at the distal end of the microtubules at 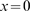. Time is defined with 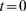 corresponding to the moment the virus capsid escapes the actin layer and attaches to a microtubule-associated motor. The viral material is biochemically processed through a chain of intermediate states 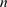 with rate 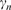, while simultaneously carried by microtubule-associated molecular motors moving at velocity 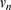 towards the nucleus at 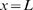. During transport, the virus particle degrades with total rate 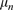. After microtubule motor assisted transport, the viral material is deposited into a perinuclear (PN) region of thickness 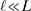, from which it enters the nucleus with rate 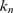. Only viral particles that enter the nucleus in state 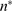 lead to a productive infection.

Here, we develop a stochastic model describing the transport, transformation, and degradation of viral material in the host cell cytoplasm. Once inside the cell, the capsid of a newly fused enveloped virus encounters a dense actin-rich cortical region that hinders its diffusion [8]. Transport to the nucleus is mediated by microtubules that penetrate this actin-rich layer, creating a highway through the cytoplasm. We thus consider an effective one-dimensional viral motion along microtubule tracks that extend from the cell periphery at 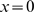 to the perinuclear (PN) region near 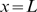, as shown in Fig. 1. The capsid is assumed to bind to microtubule-associated motors (such as dynein) at 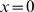 before it is convected towards the microtubule organizing center, near the perinuclear (PN) region. Processed viral material is then deposited in the thin PN layer before being transported across nuclear pores [3].

We focus on viral dynamics after attachment to the microtubule-associated motor. Viral material initially at 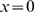 undergoes a series of transformations among 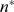 long-lived, distinguishable states while simultaneously being convected by motors with velocity 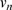. Starting from a specified initial state denoted by 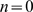, a series of irreversible transformations, such as capsid disassembly or reverse transcription, take place from state 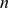 to state 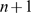 until the infective state 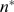 is reached. We assume that only viral material entering the nucleus in state 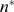 can lead to incorporation into the host genome and productively infect the cell. Since transitions are assumed to be sequential, the initial state 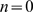 may correspond to any viral intermediate that can be quantitatively detected. For example, the “initial” state at time zero in our Markov chain might correspond to the state where minus-strand DNA syntehsis has just been completed within the reverse transcription process.

The time evolution for the probability density 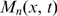 of finding microtubule-associated viral particles in state 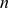, between positions 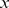 and 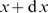, at time 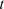, is described by the transport equation
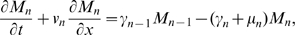
(1)where 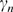 is the rate of irreversible transition from state 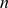 to state 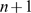, with 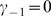. For many viruses such as HIV, fusion and initial entry are rate limiting steps and the density of virus particles in the cytoplasm is low, allowing us to consider independent particles. Furthermore, a typical dynein motor has high processivity and a run length of approximately 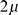
m [28], which is comparable to the length of the microtubules spanning the radius of a typical T-cell. Since backtracking and detachment/reattachment of high-processivity motors is rare, we model the viral transport as a purely convective process, with an effective velocity 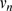 representing an average over the intrinsic molecular motor velocity and the zero drift velocity when the virus-motor complex is occasionally stalled or randomly diffusing in the cytoplasm. However, detachment of the *reverse transcriptase* (RT) machinery from the complex and general degradation, represented by 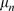, are considered irreversible. Since further transformations beyond state 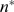 do not lead to productive infections and are equivalent to degradation, we can also set 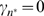 but use an appropriate degradation 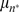. Our initial condition is 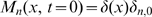.

The boundary condition at 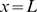 is derived by introducing a PN layer of thickness 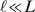 at the end of the microtubule where motors unload their viral cargoes [3], [14]. Within this thin layer, 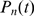, the density of viral material in state 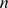, is assumed uniform so that the total amount of viral material in the PN layer, 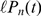, obeys
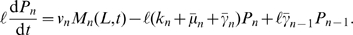
(2)Here, 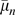 and 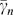 are the degradation and transformation rates specifically within the PN region. The infection probability also depends on the nuclear import rate 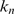, which is a function of the biochemical composition of the 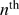 intermediate, the nuclear pore density and structure [16], and possibly chaperones that actively transport material into the nucleus [30]. Although perinuclear material at stage 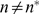 may also enter the nucleus, incompletely processed genetic material is usually not viable for integration with the host DNA [9], [22]; nuclear entry of premature intermediates can be considered to be equivalent to degradation in the PN region. Both probability densities, 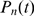 and 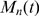 have units of probability per unit length and all of our results will be independent of 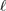 in the 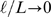 limit.

Eqs. 1–2, along with the initial condition 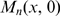, are the basic equations of our model. Since the productive infection probability will be proportional to the nuclear entry probability, we construct the probability 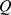 that nuclear entry eventually occurs by time-integrating the flux 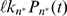 of the intermediate 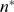 into the nucleus:
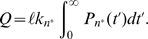
(3)Upon defining the Laplace transform 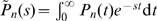, we can express the nuclear entry probability as 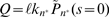. To solve for 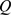, we first take the Laplace transform of Eq. 1 and solve for 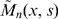:
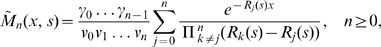
(4)where 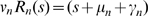 for 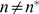 and 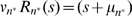. Equation 4 represents an explicit expression for the Laplace-transformed 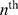
- state viral particle probability density at position 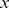 in the cytoplasm. Substitution of 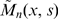 into the Laplace-transform of Eq. 2 allows us to solve for 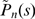 and hence 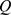:
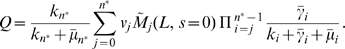
(5)Each term of the sum in Eq. 5 represents the probability of arrival of the virus particle to the PN region in state 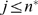, and its subsequent conversion to state 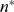 before being able to enter the nucleus. Equation 5 is our main mathematical result.

Because the exact biochemical fate of viral material within the PN layer is not well established, we will explore the consequences of our model in two limits. Since the PN region can be dense with actin meshwork, and is not traversed by microtubules, degradation and transformation rates may be negligible compared to those in the cytoplasm. This may occur, for instance, because relevant proteins such as proteasomes are too large to penetrate the dense actin meshwork near membrane-bound compartments such as the nucleus [31]. In this case 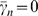 in Eq. 2, and the nuclear entry probability is simply 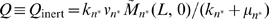, independent of 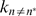 since only material that reaches the PN region at state 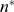 can enter the nucleus. In the opposite limit of a perinculear layer where all transformations and degradations rates are unhindered, 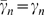, 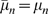, and the nuclear entry probability is denoted 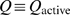.

Our equation for 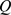 can easily be modified to include all viral states 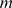 that are not microtubule bound and have 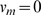. In such cases, Eq. 5 for 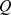 is modified by summing terms only over those states that move (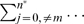) and by multiplying the resulting expression by the probability 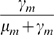 that the virus material survives each of the nonmoving states 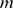.

In our subsequent analysis of Eq. 5, we will assume that all states 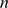 are transported velocities 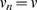 if they are microtubule-bound and convected by a single molecular motor. We will assume that a shedding virus particle will not sufficiently change its drag as to affect its transport velocity. At typical convection speeds on the order of 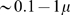
m/sec, an intact viral capsid of diameter 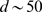
nm in cytoplasmic fluid of viscosity 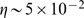
Pa imparts a drag force 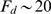
fN which is a negligible fraction (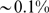) of the typical stall force of the dynein motor. Besides changing changing hydrodynamic size, different viral states 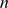 may carry inherently different motor detachment rates, changing the ratio of convective to diffusive transport. Therefore, it is possible that different states 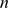 are described by different effective transport velocities 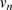. However, as long as the motor processivities are high, and convection along microtubules is more prevalent than cytoplasmic diffusion, the effective velocity can be approximated as state-independent. The only possibility of a state-dependent velocity 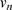, which we neglect for the sake of mathematical simplicity, is if motors of different velocity or stalling frequency are interchanged during the viral maturation process.

Table 1 below summarizes all variables and parameters defined in our model. Throughout our study, time and length units are seconds and microns, respectively. Bounds for the parameter ranges are estimated from the literature and from physical considerations. Since the definition of the states 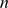 is widely varying, from different levels of capsid disassembly, to different stages of the reverse transcription process, the parameters are effective rates that can represent different processes. For example, even though motor velocities are 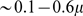
m/s, recent experiments imply that RT detaches and reattaches a number of times during the entire reverse transcription process [29]. In this scenario, both the effective transformtion rates 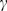 and velocity 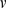 associated with reverse transcription may be significantly reduced from the nucleotide addition rate and the motor velocity, respectively. Nonetheless, we will demonstrate (cf. Supporting Information) that the qualitative features of our model arise for a very wide range of parameters and is insensitive to details.

**Table 1:** Variable and parameter definitions.

Variable or parameter	Physical definition	Units and typical values
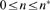	state of the viral material	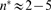
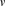	effective velocity	0.01–0.6 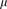 m sec 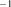
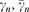	transformation rates	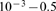 sec 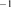
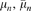	decay rates	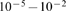 sec 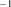
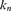	nuclear entry rates	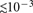 sec 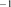
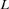	cytoplasmic distance	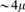 m
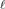	PN layer thickness	0.05 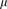 m
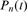	probability density in PN layer	length 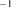
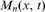	probability density at position 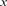	length 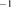
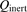	entry probability, inert PN 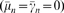	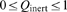
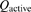	entry probability, active PN	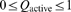

The entire infectivity process includes not only transport to the nucleus but also the subsequent stages of DNA integration and daughter virion assembly. Since the total infection probability is a product of the efficiencies of each of these processes, it will be proportional to the nuclear entry probability 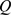 that we calculate, and to the other post nuclear penetration events that also contribute to replication efficiency losses. Finally, it is important to realize that our model of 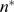 intermediates can be applied to any experimentally probed subset of all the intermediate steps of the entire, sequential infection process. Therefore, the state 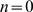 can correspond to any initially observed state if one is interested in the nuclear entry probability conditioned on starting from the specified intermediate.

## Results and Discussion

Eqs. 5 and 4 show that 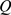 always decreases with increasing degradation rate 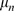. The entry probability 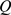 also increases with 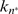 and decreases with 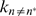. However, for various fixed decay rate patterns 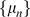, 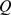 can depend on the transformation rates 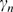 in unexpected ways. Therefore, we first explore in detail how 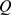 depends on the transformation rates 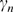.

The essence of our model is captured by considering the kinetics of a small number of longest-lived intermediate states that control the entry probability 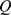. For example, 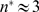 may correspond to the early, middle, and late stages of reverse transcription in HIV, as detectable by quantitative PCR [32]. For simplicity, we first assume only one transformation step, 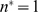, is required for productive infection. The entry probability from Eq. 5 is
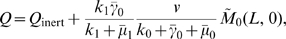
(6)where
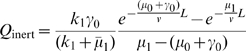
(7)is the entry probability when perinuclear transformations do not occur (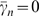). For simplicity, we assume that 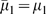. Mathematically, 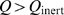; physically, this inequality arises because in the active case, immature perinuclear material at stages 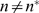 can transform to state 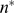 within the PN layer, enter nuclear pores, and add to 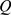. This is not possible in the inert case where perinuclear material at stage 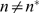 cannot contribute to infection.

**Figure 2:**
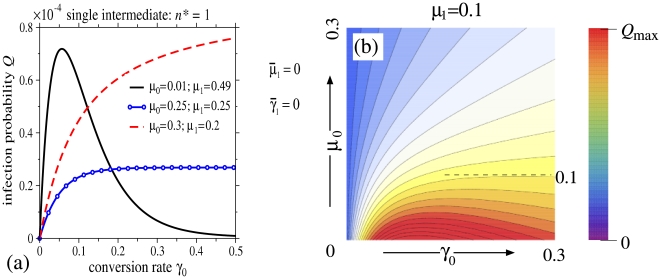
Entry probability 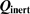 for viruses with a single intermediate step (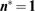). 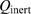
 is shown as a function of 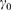 for different values of 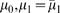. Parameters are 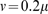
m/s, 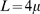
m, 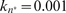
s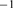. Henceforth, all parameters will be defined with time and length expressed in units of seconds and microns, respectively. (a) Optimal conversion rates exist only if 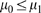, as shown by the solid curve for 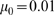 and 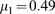. When 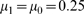 (circles), 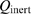 saturates as a function of 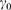, while for 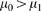 (dashed) 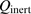 increases monotonically. When 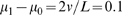, 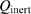 is maximized exactly at 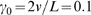. Panel (b) shows 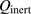 as a function of 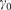 and 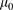 for 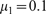. Values of 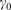 that yield local maxima in 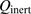 exist only if 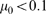.

As expected, Eq. 7 predicts that 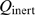 monotonically decreases with increasing 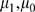 and increases with 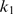. However, 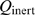 can be a non-monotonic function of the initial conversion rate 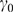, depending on values of the other parameters, as indicated in Fig. 2(a). If 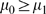, 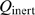 is a monotonically increasing function of 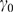, while if 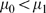, 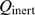 can have a maximum as a function 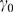. This result can be understood physically. If 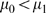, survival would be increased by decreasing the conversion rate 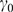 from state 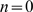 to state 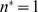 allowing a longer life-span in the less degradative state 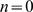. However, viral material must eventually convert to state 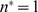 within the microtubule travel time 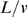 for the infection to be productive. These opposing constraints for the conversion rate lead to an intermediate value of 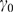 which maximizes 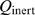. This maximum does not arise when 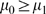 because there is no survival benefit for viral material to stay in the unprocessed stage at 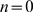. Experimental evidence consistent with local maxima in 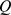 arises, for example, in the protein TRIM5 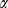 which has been shown to restrict viral infection by *accelerating* the uncoating of retroviral capsids (increasing, say, 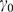) [17], [18].

**Figure 3:**
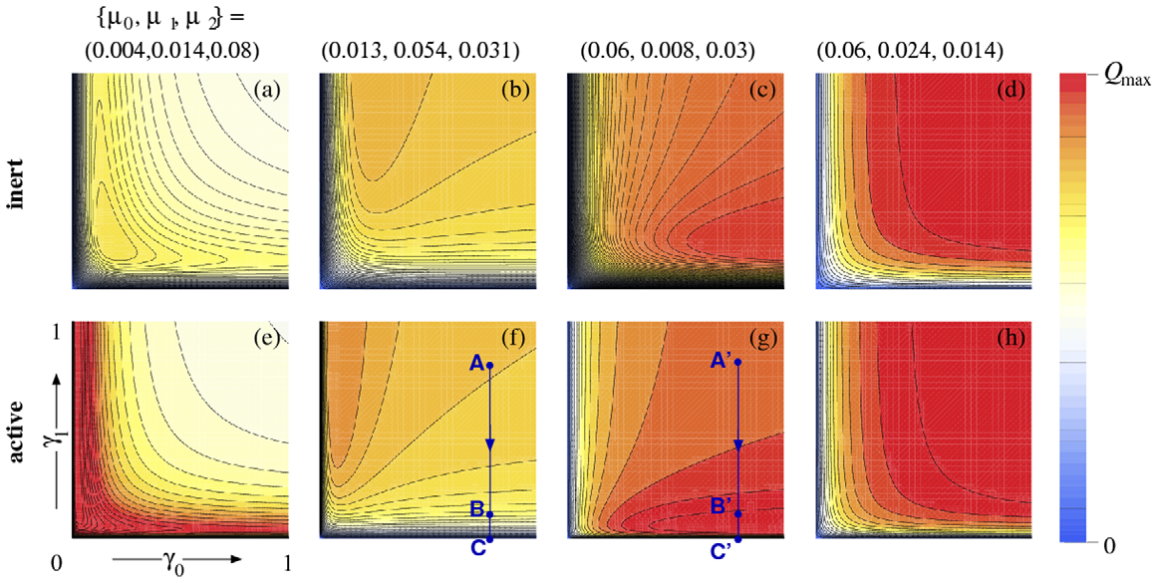
Entry probability 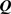 as a function of 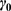 and 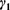 for 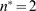. The motor velocity, microtubule length, and nuclear import rate are set to 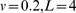 and 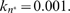 Four sets of 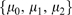, with fixed 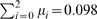, are explored. For (a–d), an inert PN region is considered (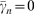). (a) When 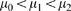 (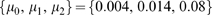), a local maximum in 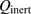 arises. (b) When the interior degradation rate 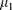 is largest, a maximum in 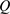 as a function of 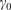 arises for large 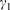. (c) When the initial and final states have larger degradation rates, a maximum as a function of 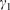 arises for large 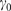. (d) For decreasing degradation rates (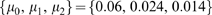), 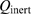 monotonically increases in both 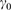 and 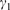. The analogous infection probabilities 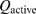 for active PN regions (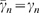 and 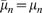) are shown in (e–h). The maxima that arise are generally sharper here than in the corresponding inert cases. The hypothetical strains depicted by (f) and (g) have different degradation phenotypes 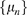, but respond differently to a 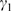-reducing antiviral. In (f), 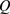 decreases from 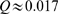 at point **A** (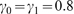) to 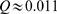 at point **B** where 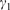 has been reduced to 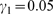. However, in strain (g), reducing 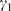 by the same amount *increases* 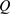 from 0.017 to 0.021. Only at points **C**, when 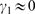 is 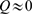 and infection suppressed.

The behaviors described above can be generalized to larger 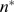. Regardless of the number 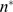 of intermediates, 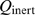 is a decreasing function of 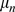 and an increasing function of 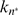. We can explore the dependence of 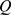 on transformation rates 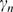 under a wide range of scenarios by considering various decay/degradation rate sequences 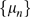. As above, for simplicity we assume that degradation rates within the PN region are the same as in the cytoplasm: 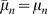. Figure 3 shows the entry probabilities for 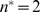 corresponding to four qualitatively different sequences 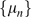 that: monotonically increase, contain a maximum, contain a minimum, and monotonically decrease.

For monotonically increasing 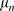 (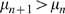), optimal values of the conversion rates 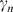 that yield a local maximum in 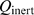 arise. If the sequence of 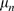 rates is not monotonically ordered, then saddle points, or maxima in 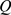 as a function of one of the 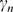, will generally arise. These are illustrated in Fig. 3(a–d) for 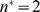, under various degradation sequences that sum to 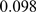. Only in the monotonically decreasing case 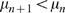 is 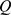 monotonic in all 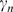, and does not exhibit a local maximum. As discussed earlier, when considering a biochemically active PN region, we consider the case 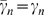 in Eq. 2. When 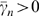, additional terms arise in Eq. 5 that increase the total infectivity 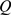. Figs. 3(e–h) show 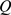 at the same parameter values used to determine 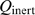 in Figs. 3(a–d), except that 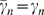 and 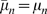. The presence of an active layer yields much sharper and more intense maxima in 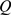 as a function of the transformation rates 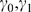. Figs. 3(a), (b) and (e) show infection probabilities that can decrease with increasing 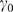. Such phenomena has been observed when TRIM5 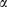 activity is increased [17], [18], and in nucleocapsid mutations that cause premature reverse transcription [20]. Both of these biological examples increase the initial transformation rates, yet decrease overall infectivity. Furthermore, mutational studies have shown that an optimal capsid stability is also required for maximal reverse transcription and productive infection [17], [19], [20], [23]. This suggests an optimal value also arises for the transformation rate associated with capsid disassembly, which is exhibited by the mathematical model.

Although any of the four pictured degradation sequences, or more complicated ones for larger 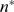, are potentially realizable, there is direct evidence for a maximum in the 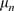 sequence for intermediate states of reverse transcription. In particular, Thomas, Ott, and Gorelick [10] measured a high-low-high pattern of step-wise efficiencies for early, middle, and late stages of reverse transcription. This pattern corresponds to a low-high-low pattern for the combination of parameters 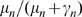. In Figs. 3(b) and (f), we assumed the correspondingly low-high-low structure for 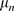 estimated from measured transformation efficiencies [10] and show how 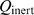 and 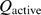 behave as 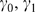 are varied.

In light of this comparison, the structure of our infection probability 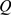 suggests an alternative conceptual guide to the development and administration of certain classes of anti-retroviral drugs such as reverse transcriptase (RT) inhibitors that act to reduce rates of subsequent transformations (lowering say, 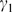). If viral complexes become less susceptible to degradation as they progress towards the nuclear entry competent stage such that 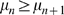, then viral infectivity will be decreased upon lowering any transformation rate 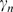 such as by the administration of the RT inhibitor, as can be seen from Fig. 3(d) and (h). On the other hand, if viral intermediate states are more susceptible to degradation such that 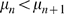, optimal theoretical values of 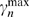 exist such that 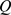 is maximized. Intrinsic transformation rates 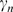 may not necessarily be near the theoretical optimal values 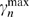.

The complex parameter dependence of the infection probability 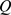 also suggests a subtle interpretation of “hidden” mutations that confer drug resistance to antivirals such as RT inhibitors [33]. In our model, genetic mutations that induce molecular changes unrelated to those that affect drug binding may still impart a more global, transport-dependent drug resistance. Specific mutations that change the transport properties (in addition to changing 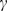) are known to exist and are also known to interact with each other in unexpected ways. For example, the M184V and L74V mutations are known to decrease RT processivity by increasing RT detachment [34], [35], which is equivalent to increasing specific 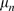 in our model. Qualitatively, a mutational change in 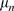 can be described by, for example, a shift of the entry probability from that shown in Fig. 3(f) to the one in Fig. 3(g). The administration of an antiviral that hinders RT on a “wild-type” viral strain (f), may be represented by **A** 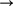 **B**. A drug-resistant mutation that affects the degradation rates alone (through, *e.g.*, decreased RT processivity) would change the 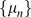 scenario such that the system would shift from point **B** to point **B′** in Fig. 3(g). Cessation of drug treatment would then bring the system to point **A′**, which has a lower entry probability that the original, untreated “wild-type” strain at point **A**.

Many mutations also have complex interactions that in certain combinations, can resensitize the virus, recovering some amount of drug susceptibility. For example, the L74V and K65R mutation together can resensitize HIV-1 to the NRTI zidovudine (AZT) [34]. Many thymidine analog mutations (TAMs) can also “interact” with the M184V mutation to increase susceptibility to AZT, stavudine, and tenofovir [36], [37]. Such complex mutation interactions may be explored by considering variations in the 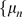, 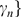 parameter space of our transport-based model. For example, if a drug-resistant strain is qualitatively described by the nuclear entry function depicted in Fig. 3(g), and an additional mutation decreases the RT processivity (such as the M184V or L74V mutations [34], [35]) the virus is now resensitized to drugs that decrease 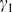.

**Figure 4:**
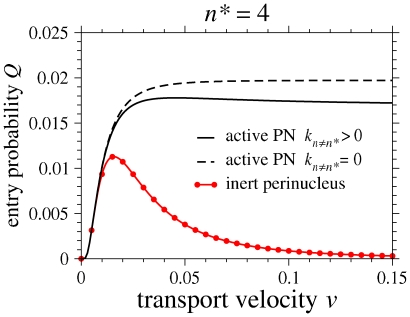
Entry probabilities as a function of microtubule transport velocity 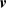. The entry probability 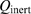 associated with an inert PN region (circles) is low, but exhibits a maximum. When perinuclear transformations occur (dashed curve) the virus material converts to infection-competent species 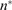 while it is waiting in the PN region, increasing 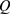. However, the maximum in 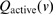 disappears. When nuclear import of infection-incompetent species are also allowed 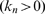, effective degradation increases through unproductive entries, and the maximum in 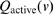 can reappear. These curve were generated using representative parameters 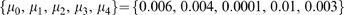, 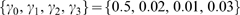, 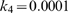, and 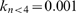.

In addition to the rich infection behavior possible under variations in 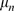 and 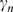, we identify another key parameter 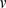, the speed at which viral material is transported along the microtubules. Dynein motors, which transport viral cargo to the nucleus, can move with a range of velocities 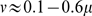
m/sec, with higher ATP concentrations leading to higher motor velocities [38], [39]. An analysis of 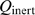 as a function of 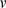 shows that when 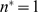, 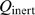 is maximized at 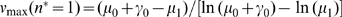. For 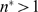 optimal speeds that maximize 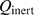 as a function of 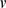 also arise and can be found numerically; entry probabilities as a function of 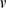 are shown in Fig. 4 for 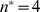. The existence of optimal transport velocities for 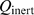 results from two opposing effects: high velocities do not provide sufficient time for the virus particles to reach the infective state 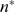, while low velocities increase exposure to cytoplasmic degradation. The behavior of 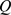 as a function of 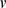 is also sensitive to perinuclear transformation and nuclear entry rates. In the case of an active PN layer where only material at state 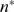 can enter the nucleus (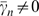 and 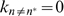), the entry probability 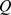, although greater than 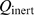, becomes a monotonically increasing function of 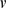 and loses its maximum as shown by the dashed curve in Fig. 4. In this case the under-processed viral material simply waits in the PN layer, eventually converting to state 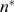, whereupon nuclear entry is possible. Thus, there is no penalty in reaching the PN region in state 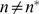, and the entry probability 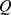 increases with 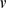 since higher velocities allow the virus to better escape cytoplasmic degradation with the possibility of completing biochemical processes upon arrival at 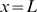.

Now, suppose that viral material arriving in the PN region at state 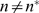 can enter the nucleus at rate 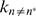, but still without leading to productive infection. The entry of this improperly processed material does not contribute to 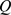. All else being equal, allowing the nuclear entry of incomplete material yields a smaller entry probability 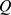 compared to the previous case of an active perinucleus with 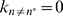. Here, the maximum in 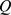 as a function of 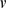 is recovered, as shown by the solid black intermediate curve in Fig. 4. As in the case of an inert PN layer, a maximizing transport velocity 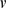 arises, albeit for different biochemical reasons. While higher transport velocities 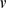 allow for less degradation in the cytoplasm, reaching the PN region in a state 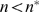 increases the probability of unproductive nuclear import.

Our model illustrates how infection probabilities can be controlled by varying motor velocities, by adjusting *e.g.*, cellular ATP concentration. Although varying ATP concentrations may also affect both the degradation and transformation rates of the infection process, it does suggest in principle that varying motor velocities might be used to probe viral kinetics within the PN region. If higher velocities 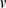 always lead to higher measured infection probabilities, the PN region is active and only completely processed material can enter the nucleus. As shown by the dashed curve in Fig. 4, an increasing 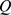 as a function of 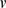 corresponds to the case of 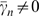 and 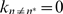. On the other hand, if a maximum is observed as a function of 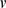, the PN region is either inert (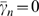), or is active but facilitates nuclear entry of incompletely processed viral material (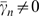, 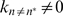). To distinguish between these two cases we propose in the Supporting Information, another independent measurement: the mean first time of productive infection, conditioned on realization of a productive infection (cf. Fig. 1 of File S1). However, the qualitative features of the computed nuclear entry probability, 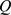, are fairly robust with respect to interdependences of our parameters. For example, Fig. 2 of File S1 shows that qualitatively reasonable velocity and transformation rate dependences on ATP concentration can lead to optimal entry probabilities at intermediate ATP concentrations. Finally, we note that all results depend on transport velocity and cell radius through the combination 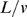. Therefore, the qualitative dependences on 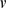 also hold for 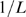; specifically, for a fixed transport velocity, we expect that for a host cell with an inactive perinucleus, a particular host cell radius 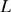 that optimizes nuclear entry probability may arise.

The rate parameters in Table 1 and used to generate our curves are very rough estimates inferred from the literature on infectivity assays. Recent experiments show that many of these rates vary significantly depending on cell type, cell state, and on assay preparation [15]. As a result, the above values are indicative and may change depending on the particular HIV sample under investigation. For example, initial delays up to the order of hours may occur in the peripheral cytoskeleton, adding to an offset in the zero of time at which the viral material starts to be transported by microtubules. Prolonged delays have been measured between the completion of RT and nuclear entry [15]. These delays appear to vary in certain cell types, and are incorporated in our model through the nuclear entry rate 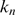. However, our qualitative finding of maxima in nuclear entry probability 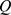 is insensitive to the magnitudes of 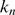. We also demonstrate in the Supporting Information how more complicated parameter interdependencies may preserve the mathematical structure and qualitative features of our results. Specifically, we show that if RT transformation rates 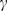 and the transport velocity 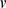 are both linearly dependent on ATP concentration, a maximum nuclear entry probability arises at intermediate values of ATP.

Summarizing, the cellular physics accompanying viral infections involves a number of complex and orchestrated biochemical steps. We have proposed a phenomenological, yet illustrative stochastic model that incorporates the basic, known processes of viral transport, degradation, conversion, and nuclear entry in the infection process. Including only these processes in our model, we find a rich dependence of the productive infection probability and the mean time to infection on cytoplasmic and perinuclear transport parameters.

In *vivo*, we expect transport rates and perinuclear properties to be evolutionarily selected according to constraints imposed by the host cell to optimize a combination of infection speed and infection probability. We explored and found parameter regimes where viral infectivity can be optimized under constraints of active or inactive PN regions and of the nuclear import kinetics. Mathematically, the infection probability 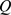, regardless of whether the PN region is inert or active, is

- a decreasing function of degradation rates 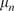 for all 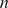 and of entry rates 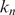 for 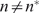;
- an increasing function of nuclear import 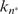;
- an increasing function of the transformation rates 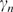 for 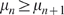 such as in Fig. 3(d) and Fig. 3(h);
- a non-monotonic function of 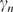 for 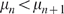. Here, optimal infectivity probabilities exist as a function of 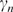 such as in Figs. 3(a–c) and Fig. 3(a–g);

Maxima in the infection probability 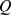 also imply that apparent drug resistant strains can arise through mutations in genes unrelated to the viral components that directly interact with the antiviral drug. For degradation rates 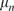 that are non-monotonic in 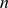, the infection probability 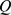 may contain maxima or saddles as a function of the 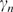. Antiviral drugs designed to simply inhibit processes (such as reverse transcription) by reducing the appropriate 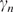, must be administered as to not increase 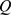. Moreover, since the rate of detachment of molecular motors from cytoskeletal filaments can be biochemically controlled [40]–[42], drugs that target the degradation structure 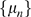 and/or transport velocity 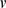 can have complex interactions with 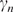-decreasing antivirals, amplifying their beneficial or harmful effects. Indeed, these “indirect” antiviral drugs (such as Gleevec) that affect transport have been shown to block vaccinia virus infection [6].

The transport-transformation model can also be used to potentially explain resensitization of antivirals. Moreover, when more than one drug is administered, cross resistance can also arise [43], [44]. Within the language of our model, two drugs that increase the same 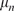, or decrease the same 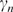 are expected to drive a cross-resistant mutation. Although drug-resistant mutants typically have overall lower replicative efficiency in absence of drug, there is recent evidence that antiviral protein resistant mutants of the bacteriophage 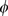
×174 can carry fitness levels above that of the wild type [45]. Although the physics of the bacteriophage infection process is different from the filament-nucleus mechanism described by our model, the mathematical structure of our transport-based model does allow for the possibility for the nuclear entry probability of a drug-resistant mutant to be lower than that of the wild-type. More refined experiments of efficiencies of reaching intermediate stages during the infection process may help to refine our genotypical understanding of drug resistance. Analysis of our model also potentially provides a guide for probing the nature of the dynamics within the PN region, based on measurements of the first infection times after initial entry into the host cell. First passage times between viral material states, as well as the resulting biophysical implications are discussed in the Supporting Information.

## Supporting Information

File S1 Supporting Information file with figures and legends.
(0.06 MB PDF)

## References

[1] Nowak S, Chou T (2009). Mechanisms of receptor/coreceptor-mediated entry of enveloped viruses.. Biophys J.

[2] Chou T, D'Orsogna MR (2007). Multistage adsorption of diffusing macromolecules and viruses.. J Chem Phys.

[3] Fields BN, Knipe DM, Howley PM, Griffin DE (2007). Fields Virology, 5^th^ Edition,.

[4] Vodicka MA (2001). Determinants for lentiviral infection of non-dividing cells.. Som Cell Mol Genet.

[5] Greene WC, Peterlin BM (2002). Charting HIV's remarkable voyage through the cell: Basic science as a passport to future therapy.. Nature Med.

[6] Greber UF, Way M (2006). A superhighway to virus infection.. Cell.

[7] Anderson JL, Hope TJ (2005). Intracellular trafficking of retroviral vectors: obstacles and advances.. Gene Therapy.

[8] Sodeik B (2000). Mechanisms of viral transport in the cytoplasm.. Trends in Microbiology.

[9] Bukrinsky M (2004). A hard way to the nucleus.. Mol Med.

[10] Thomas JA, Ott DE, Gorelick RJ (2007). Efficiency of Human Immunodeficiency Virus Type I Postentry Infection Processes: Evidence against Disproportionate Numbers of Defective Virions.. J Virology.

[11] McDonald D, Vodicka MA, Lucero G, Svitkina TM, Borisy GG (2002). Visualization of the intracellular behavior of HIV in living cells.. J Cell Bio.

[12] Seisenberger G, Ried MU, Endreß T, Büning H, Hallek M (2001). Real-Time Single-Molecule Imaging of the Infection Pathway of an Adeno-Associated Virus.. Science.

[13] Lakadamyali M, Rust MJ, Babcock HP, Zhuang X (2003). Visualizing infection of individual influenza viruses.. Proc Natl Acad Sci.

[14] Arhel N, Genovesio A, Kyeong-Ae K, Miko S, Perret E (2006). Quantitative four-dimensional tracking of cytoplasmic and nuclear HIV-1 complexes.. Nat Meth.

[15] Arfi V, Lienard J, Nguyen XN, Berger G, Rigal D (2009). Characterization of the behavior of functional viral genomes during the early steps of human immunodeficiency virus type 1 infection.. J Virol.

[16] Holcman D (2008). Effective Motion of a Virus Trafficking Inside a Biological Cell.. SIAM J Appl Math.

[17] Stremlau M, Perron M, Lee M, Li Y, Song B (2006). Specific recognition and accelerated uncoating of retroviral capsids by the TRIM5*α* restriction factor.. Proc Natl Acad Sci.

[18] Perron MJ, Stremlau M, Lee M, Javanbakht H, Song B (2006). The Human TRIM5*α* Restriction Factor Mediates Accelerated Uncoating of the N-Tropic Murine Leukemia Virus Capsid.. J Virology.

[19] Yamashita M, Emerman M (2004). Capsid Is a Dominant Determinant of Retrovirus Infectivity in Nondividing Cells.. J Virol.

[20] Thomas JA, Bosche WJ, Shatzer TL, Johnson DG, Gorelick RJ (2008). Mutations in Human Immunodeficiency Virus Type 1 Nucleocapsid Protein Zinc Fingers Cause Premature Reverse Transcription.. J Virol.

[21] Dismuke DJ, Aiken C (2006). Evidence for a Functional Link between Uncoating of the Human Immunodeficiency Virus Type 1 Core and Nuclear Import of the Viral Preintegration Complex.. J Virol.

[22] Iordanskiy S, Berro R, Altieri M, Kashanchi F, Bukrinsky M (2006). Intracytoplasmic maturation of the human immunodeficiency virus type 1 reverse transcription complexes determined their capacity to integrate into chromatin.. Retrovirol.

[23] Forshey BM, von Schwedler U, Sundquist WI, Aiken C (2002). Formation of a human immunodeficiency virus type 1 core of optimal stability is crucial for viral replication.. J Virol.

[24] Yamashita M, Emerman M (2006). Retroviral infection of non-dividing cells: Old and new perspectives.. Virol.

[25] Dee KU, Shuler ML (1997). A mathematical model of the trafficking of acid dependent enveloped viruses: application to the binding, uptake and nuclear accomodation of baculovirus.. Biotech Bioeng.

[26] Smith DA, Simmons RM (2001). Models of motor-assisted transport of intracellular particles.. Biophys J.

[27] Dinh AT, Theofanous T, Mitragotri S (2005). A model for intracellular trafficking of adenoviral vectors.. Biophys J.

[28] Reck-Peterson SL, Yildiz A, Carter AP, Gennerich A, Zhang N (2006). Single-Molecule Analysis of Dynein Processivity and Stepping Behavior.. Cell.

[29] Liu S, Abbondanzieri EA, Rausch JW, Le Grice SFJ, Zhuang X (2008). Slide into Action: Dynamic Shuttling of HIV Reverse Transcriptase on Nucleic Acid Substrates.. Science.

[30] Brass AL, Dykxhoorn DM, Benita Y, Yan N, Engelman A (2008). Identification of host proteins required for HIV infection through a functional genomic screen.. Science.

[31] Luby-Phelps K, Taylor DL (1988). Subcellular Compartmentalization by Local Differentiation of Cytoplasmic Structure.. Cell Motility and the Cytoskeleton.

[32] Krogstad P, Chen ISY, Canon J, Rey O (1996). Qualitative Analysis of the Endogenous Reverse Transcriptase Reaction of HIV Type 1 Variants with Decreased Susceptibility to Azidothymidine and Nevirapine.. AIDS Res and Hum Retroviruses.

[33] Ehteshami S, Beilhartz GL, Scarth BJ, Tchesnokov EP, McCormick S (2008). Connection Domain Mutations N348I and A360V in HIV-1 Reverse Transcriptase Enhance Resistance to 3′-Azido-3′-deoxythymidine through Both RNase H-dependent and -independent Mechanisms.. J Biol Chem.

[34] Vivet-Boudou V, Didierjean J, Isel C, Marquet R (2006). Nucleoside and nucleotide inhibitors of HIV-1 replication.. Cell Mol Life Sci.

[35] Sharma PL, Crumpacker CS (1999). Decreased processivity of human immunodeficiency virus type 1 reverse transcriptase (RT) containing didanosine-selected mutation Leu74Val: a comparative analysis of RT variants Leu74Val and lamivudine-selected Met184Val.. J Virol.

[36] Naeger LK, Margot NA, Miller MD (2001). Increased drug susceptibility of HIV-1 reverse transcriptase mutants containing M184V and zidovudine-associated mutations: analysis of enzyme processivity, chain-terminator removal and viral replication.. Antiviral Therapy.

[37] Parkin N, Chappey C, Petropoulos C, Hellmann N (2003). HIV-1 reverse transcriptase mutations that suppress zidovudine resistance also increase in vitro susceptibility to tenofovir, but not stavudine.. Antiviral Therapy.

[38] Singh MP, Mallik R, Gross SP, Yu C (2005). Monte Carlo modeling of single molecule cytoplasmic dynein.. Proc Natl Acad Sci.

[39] Gao YQ (2006). A Simple Theoretical Model Explains Dynein's Response to Load.. Biophys J.

[40] Thorn KS, Ubersax JA, Vale RD (2000). Engineering the Processive Run Length of the Kinesin Motor.. J Cell Bio.

[41] Hodges AR, Krementsova EB, Trybus KM (2007). Engineering the Processive Run Length of Myosin V.. J Biol Chem.

[42] Cho C, Reck-Peterson SL, Vale RD (2008). Regulatory ATPase Sites of Cytoplasmic Dynein Affect Processivity and Force Generation Formula.. J Biol Chem.

[43] Baldanti F, Paolucci S, Maga G, Labo N, Hübscher U (2003). Nevirapine-selected mutations Y181I/C of HIV-1 reverse transcriptase confer cross-resistance to stavudine.. AIDS.

[44] Paolucci S, Baldanti F, Maga G, Cancio R, Zazzi M (2004). Gln145Met/Leu Changes in Human Immunodeficiency Virus Type 1 Reverse Transcriptase Confer Resistance to Nucleoside and Nonnucleoside Analogs and Impair Virus Replication.. Antimicrobial Agents and Chemotherapy.

[45] Cherwa JE, Sanchez-Soria P, Wichman HA, Fane BA, Members of the University of Arizona Virology Laboratory Course 2007 (2009). Viral Adaptation to an Antiviral Protein Enhances the Fitness Level to Above That of the Uninhibited Wild Type.. J Virology.

