# Supplementary material for: Optimal Cytoplasmic Transport in Viral Infections
Source: PLoS One. 2009 Dec 30;4(12):e8165. doi: 10.1371/journal.pone.0008165 (PMC2797302; doi:10.1371/journal.pone.0008165)
Supplement: File S1 — Supporting Information file with figures and legends. (0.06 MB PDF) [file pone.0008165.s001.pdf]

Supporting Information: Optimal cytoplasmic transport in viral infections

Maria R. D'Orsogna[a] and Tom Chou[b]

[a]Dept. of Mathematics, CalState-Northridge, Los Angeles, CA 91330-8313
[b]Dept. of Biomathematics & Dept. of Mathematics, UCLA, Los Angeles, CA 90095-1766

November 13, 2009

Mean entry times

Here, we outline the calculation and analysis of first passage times to nuclear entry, starting at a specified state $n = 0$ near the cell periphery. Again, the qualitative features of our model are insensitive to the details of the rate parameters, and we use the representative values employed in Figure 4.

From Eq. 2, the flux into the nucleus at time t is given by $J_{n^*}(t) = \ell k_{n^*} P_{n^*}(t)$. Upon using standard methods [1, 2], we obtain the conditional first passage time distribution $G(t)$ as the conditional probability flux

$$G(t) = \frac{J_{n^*}(t)}{\int_0^\infty J_{n^*}(t')\mathrm{d}t'} \equiv \frac{J_{n^*}(t)}{Q}. \tag{S1}$$

The mean conditional first infection time, the time it takes for nuclear entry after attaching to a microtubule *given* that the virus made it into the nucleus, is thus given by

$$T = \int_0^\infty tG(t)\mathrm{d}t \equiv -\frac{1}{\tilde{P}_{n^*}}\frac{d\tilde{P}_{n^*}}{ds}\bigg|_{s=0} = \frac{L}{v} + \tau, \tag{S2}$$

where

$$\tau = \frac{\sum_{j=0}^{n^*} \tau_j \tilde{M}_j(L, s=0) \prod_{i=j}^{n^*-1} \frac{\bar{\gamma}_i}{k_i+\bar{\gamma}_i+\bar{\mu}_i}}{\sum_{j=0}^{n^*} \tilde{M}_j(L, s=0) \prod_{i=j}^{n^*-1} \frac{\bar{\gamma}_i}{k_i+\bar{\gamma}_i+\bar{\mu}_i}}, \tag{S3}$$

and

$$\tau_j = \sum_{i=j}^{n^*} (k_i + \bar{\gamma}_i + \bar{\mu}_i)^{-1}. \tag{S4}$$

The total mean conditional first infection time, T, valid for both inert and active layers, is given by the ballistic time to reach the PN region, plus τ, the time to enter the nucleus from the PN

region. Both T and $\tau = T - L/v$ are shown in Fig. 4(b) for inert and active PN regions. For the case of an inert PN layer where $\bar{\gamma}_n = 0$ and only material that reaches $x = L$ in state n^* may enter nuclear pores ($k_{n \neq n^*} = 0$), τ simplifies to $\tau_{\rm inert}^{-1} = k_{n^*} + \mu_{n^*}$ and is independent of transition and degradation rates of states $n \neq n^*$. The perinuclear contribution τ is simply the first passage time to enter the nucleus in the presence of degradation of state n^* in the PN region. The waiting time τ changes dramatically when the PN layer is active. Here, each of the summed terms of τ in Eq. S3 represents the time τ_j needed for material in state $j \neq n^*$ in the PN region to be processed into state n^*. These arrival times are weighted by the respective probabilities of reaching the PN layer in state j as expressed in Eq. S3. It is straightforward to show that $\tau_{\rm active}$ (when $\bar{\gamma}_n > 0$) is an increasing function of v.

In the case of an active PN layer, incompletely processed material accumulates in the perinucleus, waiting to be processed. This accumulation of viral material has been observed in the case of HIV [4, 5]. Higher speeds v result in perinuclear material at very low levels of maturity, $n \ll n^*$, since not enough time has elapsed for all n^* transformations to occur and relatively more time is spent in the PN layer in states $n < n^*$. In contrast to the non-monotonic behavior of Q as a function of v, a monotonically increasing $\tau_{\rm active}$ due to perinuclear transformations, added to the ballistic time L/v remains a decreasing function of v. While the behavior of Q *versus* v alone cannot determine the transformation and entry kinetics in the PN region, experimental determination of the first passage time as a function of v (or ATP concentration), allows one to unambiguously resolve them. As shown in Fig. 1, for an inert PN region, τ is independent of v, while for an active region, τ is an increasing function of v, regardless of the structure of the nuclear entry rate k_n. Combined with the previous analysis of Q (Fig. 4) the exact fate of perinuclear material can now be determined.

Parameter Interdependences

We now consider evaluating the global effects of a simultaneous change in v and γ within our model. This particular interdependence may occur when ATP concentration, that affect both motor speed v and reverse transcription rates, is varied. It is not difficult to incorporate this scenario, given some plausible functional forms on how γ and v change with ATP concentration.

To investigate the above possibility, we focus on the $n^* = 4$ case of Fig. 4 in our manuscript. We take our estimates for $Q_{\rm inert}$ and $Q_{\rm active}$ (with nuclear entry rates $k_{n \neq n^*}$ either zero or non zero) in Eq. 4 and assume that v and one of the γ rates are changing with ATP concentration [ATP]. For simplicity, we choose a linear dependence for $v([\mathrm{ATP}])$ and $\gamma_1([\mathrm{ATP}])$, so that $v([\mathrm{ATP}]) = \delta v \times [\mathrm{ATP}]$ and $\gamma_1([\mathrm{ATP}]) = \gamma_1 + \delta\gamma_1 \times [\mathrm{ATP}]$. In this example, we let only γ_1 change, with ATP, mimicking the ATP dependence of, say, the reverse transcription step only. We keep the other rates unmodified, assuming that all previous and subsequent viral stages are independent of ATP, including capsid disassembly, represented by γ_0 , and the incorporation of post-reverse transcription proteins, and the formation of the preintegration complex, represented by γ_2 and γ_3, respectively.

As shown in Fig. S2, the overall features of Q when both v and γ are functions of [ATP] are similar to those when only v is the control parameter. It is straightforward to incorporate the effects of externally controlled parameters on other rates and velocities, as long as functional forms for these dependencies are specified.

References

[1] Redner S (2007) *A Guide to First-Passage Processes* 1st ed. Cambridge University Press.

[2] Papoulis A (1984) "Conditional Probabilities and Independent Sets" in *Probability, Random Variables, and Stochastic Processes*, 2nd ed. McGraw-Hill, New York.

[3] Seisenberger G, Ried MU, Endreß T, Büning H, Hallek M, Bräuchle C (2001) Real-Time Single-Molecule Imaging of the Infection Pathway of an Adeno-Associated Virus. *Science* 294:1929-1932.

[4] Fields BN, Knipe DM, Howley PM, Griffin DE (2007) *Fields Virology, 5th Edition*, (Lippincott Williams & Wilkins, 2007).

[5] McDonald D, *et al.* (2002) Visualization of the intracellular behavior of HIV in living cells. *J. Cell Bio.* 159:441-452.

Figure Legends

Figure 1: Mean productive infection times computed using the same rate parameters as those used in Fig. 4. These parameters give conditional infection times on the order of tens of minutes, consistent with single virus particle tracking experiments [3]. The thin curves are the delay times τ (Eq. S3) associated with the PN region. In the case of an active PN layer, τ is an increasing function of v: at higher velocities, viral material reaches the PN layer at less mature stages, and longer times are required to reach the entry competent stage n^*. On the other hand, for an inert layer τ is constant since only mature viral compounds can enter the nucleus and the waiting time is simply $(\mu_{n^*} + k_{n^*})^{-1}$. Besides the cytoplasmic time $T = L/v + \tau$ the total viral infection time includes the initial waiting time within the peripheral actin as well as nuclear processing and budding times, which are not modeled in this work.

Figure 2: Entry probabilities $Q_{\rm inert}$ and $Q_{\rm active}$ with nuclear import rates set to zero ($k_n = 0, n \neq n^*$) and with non-zero nuclear import rates $k_n \neq 0, n \neq n^*$. (a) Entry probabilities as a function of [ATP] for $\delta v = 0.5\mu$m/(mM sec) and all γ_n independent of [ATP]. All other parameters are those used in Fig. 4. (b) Here, we assume that both convection speed v and transformation rate γ_1 increase linearly with ATP concentration via the proportionalities $\delta v = 0.5\mu$m/(mM s) and $\delta\gamma_1 = 0.006$ (sec mM)$^{-1}$. For these and a wide range of other parameters, there is no significant qualitative difference between the two scenarios. This shows that our qualitative conclusion of optimal values of transformation rates γ is common within our mathematical structure.
